# Progenitor-derived hepatocyte-like (B-13/H) cells metabolise 1′-hydroxyestragole to a genotoxic species via a SULT2B1-dependent mechanism

**DOI:** 10.1016/j.toxlet.2015.12.010

**Published:** 2016-01-22

**Authors:** Philip M. Probert, Jeremy M. Palmer, Wasma Alhusainy, Aimen O. Amer, Ivonne M.C.M. Rietjens, Steven A. White, David E. Jones, Matthew C. Wright

**Affiliations:** a Institute Cellular Medicine, Newcastle University, Newcastle Upon Tyne, United Kingdom; b Division of Toxicology, Wageningen University, Tuinlaan 5, 6703HE Wageningen, The Netherlands

**Keywords:** CYP, cytochrome P450s, DEX, dexamethasone, E-3′-*N*^2^-dGuo, *N*^2^*-*(*trans*-isoestragol-3′-yl)-2′-deoxyguanosine, GSTs, glutathione S transferases, OTM, olive tail moment, PAPSS, 3′-phosphoadenosine 5′-phosphosulphate synthase, PCP, pentachlorophenol, PNP, ρ-nitrophenol, SULT, sulphotransferase, UDPGTs, UDP-glucuronyl transferases, Estragole, Sulfotransferase, Liver, Genotoxicity, Comet

## Abstract

Rat B-13 progenitor cells are readily converted into functional hepatocyte-like B-13/H cells capable of phase I cytochrome P450-dependent activation of pro-carcinogens and induction of DNA damage. The aim of the present study was to investigate whether the cells are also capable of Phase II sulphotransferase (SULT)-dependent activation of a pro-carcinogen to an ultimate carcinogen. To this end we therefore examined the bioactivation of the model hepatic (hepato- and cholangio-) carcinogen estragole and its proximate SULT1A1-activated genotoxic metabolite 1′-hydroxyestragole. Exposing B-13 or B-13/H cells to estragole (at concentrations up to 1 mM) resulted in the production of low levels of 1′-hydroxyestragole, but did not result in detectable DNA damage. Exposing B-13/H cells – but not B-13 cells – to 1′-hydroxyestragole resulted in a dose-dependent increase in DNA damage in comet assays, confirmed by detection of *N*^2^*-*(*trans*-isoestragol-3′-yl)-2′-deoxyguanosine adducts. Genotoxicity was inhibited by general SULT inhibitors, supporting a role for SULTS in the activation of 1-hydroxyestragole in B-13/H cells. However, B-13 and B-13/H cells did not express biologically significant levels of SULT1A1 as determined by qRT-PCR, Western blotting and its associated 7-hydroxycoumarin sulphation activity. B-13 and B-13/H cells expressed – relative to intact rat liver – high levels of SULT2B1 (primarily the b isoform) and SULT4A1 mRNAs and proteins. B-13 and B-13/H cells also expressed the 3'-phosphoadenosine 5′-phosphosulphate synthase 1 required for the generation of activated sulphate cofactor 3′-phosphoadenosine 5′-phosphosulphate. However, only B-13/H cells expressed functional SULT activities towards SULT2B1 substrates DHEA, pregnenolone and 4 methylumbelliferone. Since liver progenitor cells are bi-potential and also form cholangiocytes, we therefore hypothesised that B-13 cells express a cholangiocyte-like SULT profile. To test this hypothesis, the expression of SULTs was examined in liver by RT-PCR and immunohistochemistry. SULT2B1 – but not SULT1A1 – was determined to be expressed in both rat and human cholangiocytes. Since 1′-hydroxyestragole exposure readily produced DNA injury in B-13/H cells, these data suggest that cholangiocarcinomas generated in rats fed estragole may be dependent, in part, on SULT2B1 activation of the 1′-hydroxyestragole metabolite.

## Highlights

• B-13/H cells are null for functional SULT1A1 but express functional SULT2B1 activity.
• B-13/H cells activate 1′ hydroxyestragole to a genotoxic metabolite.
• SULT2B1 is expressed in cholangiocytes in rat and human liver.
• B-13/H cells express a cholangiocyte pattern of sulphotransferase (SULT) expression.
• Tumours in estragole-exposed rats may be dependent on cholangiocyte SULT2B1.

## Graphical abstract

**Figure:**
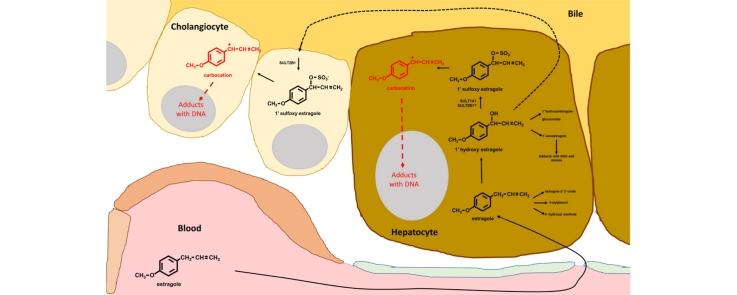

## 1 Introduction

Genotoxicity refers to the process by which chemicals or other agents damage DNA, leading to an alteration in DNA structure, information content or segregation (EFSA, 2011). In the absence of effective repair, genotoxicity is the first step in the initiation of mutagenesis and potentially of carcinogenesis (EFSA, 2011). For drugs and chemicals therefore, knowledge of any potential genotoxic activity is essential in any assessment of safety.

A variety of in vitro assays have been developed to screen for potential genotoxic chemicals. Since chemicals are very often indirect genotoxins and require metabolism to display their genotoxic activity, many assays (e.g. Ames tests, micronucleus tests) include the option of addition of liver extracts rich in enzymes that activate indirect genotoxins (e.g. S9 extracts). Hepatocytes provide an opportunity to examine potential chemical genotoxicity in cells that express a complex complement of xenobiotic metabolising enzymes (activating and de-activating) in an intact cell-based model. However, human hepatocytes are in short supply (and are of variable quality) and hepatocytes from animals require donors. Primary hepatocytes also rapidly de-differentiate in culture (Wallace et al., 2010a).

This group has therefore investigated the utility of a male rat progenitor B-13 cell as a cost-effective donor-free hepatocyte source and model system to screen for both chemical toxicity and genotoxicity Marek et al., 2003, Wallace et al., 2010a, Probert et al., 2014a, Fairhall et al., 2013). The B-13 cell line is readily expandable on simple plastic and basic culture media and in response to a single glucocorticoid hormone treatment, trans-differentiates into a non-replicative hepatocyte-like (B-13/H) cell expressing near normal levels of many cytochrome P450 (CYP) enzymes in a masculine pattern (Marek et al., 2003, Wallace et al., 2010b, Probert et al., 2015). The CYPs are functional, metabolise probe substrates and activate CYP-activated hepatotoxins (Marek et al., 2003, Wallace et al., 2010b, Fairhall et al., 2013). Furthermore, the majority of the CYP induction pathways appear to be intact and in the B-13/H phenotype, induction and metabolic activation of several genotoxins (benz[α]pyrene, PhIP, aflatoxin B1) has been demonstrated (Probert et al., 2014a). In contrast to primary hepatocytes, B-13/H cells also remain differentiated on plastic for several weeks (Marek et al., 2003). The B-13 cell system therefore offers a potential model system in which to study chemical toxicity and genotoxicity. The added value of this system is that animal donors are not required, it is relatively easy to generate genetically-modified variants of the cell line (e.g. modulate expression of genes predicted to impact on toxicity/genotoxicity) and production of large quantities of cells is cost effective when compared to less functional stem cell-derived hepatocytes (Probert et al., 2015).

**Fig. 1:**
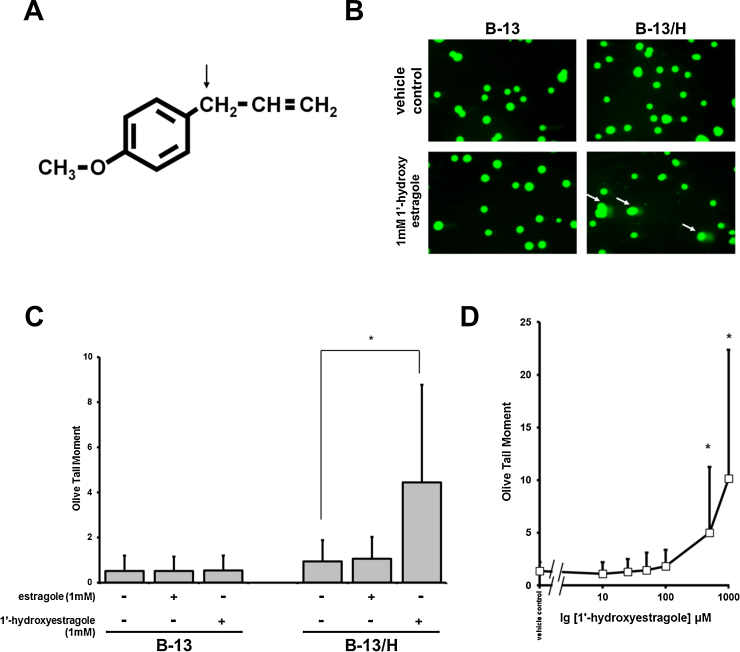
1′-hydroxyestragole but not estragole caused dose-dependent DNA damage in B-13/H cells. A. Structure of estragole. Arrow indicates site of cytochrome-mediated 1′ hydroxylation. B. Fluorescent photomicrograph of B-13 and B-13/H cell DNA following 1′-hydroxyestragole treatment and comet assay. DNA was stained with SYBR gold and visualised using the FITC filter. C. DNA damage in B-13 and B-13/H cells treated as indicated after 24 h. *indicates significantly different. D. DNA damage in B-13/H cells treated with different concentrations of 1'-hydroxyestragole for 24 h. *indicates significantly different to vehicle treated cells.

Since some genotoxins require phase II enzyme systems for activation to a proximate genotoxin, the B-13 platform was therefore examined for its ability to identify a genotoxin requiring phase II activation. Estragole is an alkenylbenzene (for structure see Fig. 1A) naturally present in herbs such as tarragon, basil and fennel (EU-SCF, 2001). Estragole is extensively metabolised to non-toxic metabolites (Alhusainy et al., 2012). However, monoxygenation at the 1′ position to produce 1′-hydroxyestragole by predominantly CYP1A2 and CYP2A6 in man (Jeurissen et al., 2007), followed by conjugation with sulphate by suphotransferases (SULTs) leads to the generation of 1′-sulphoxyestragole, which is unstable and forms a reactive carbocation (Phillips et al., 1981, Gardner et al., 1996, Jeurissen et al., 2007). Estragole has been shown to cause multiple cholangiocarcinomas and hepatocellular adenoma in the liver of male rats (Miller et al., 1983, Bristol, 2011) with reactive metabolite formation in the liver recently confirmed (Ding et al., 2015).

A screen for phase II enzymes in B-13 cells indicated that there is an up-regulation in the expression of these genes in B-13/H (compared with B-13 cells) and that B-13/H cells expressed 5 (from a total of 14) SULTs, 5 (from a total of 8 hepatic) UDP-glucuronyl transferases and 15 (from a total of 21) glutathione S transferases transcripts (Probert et al., 2014a). We therefore hypothesised that B-13/H cells are capable of SULT-mediated sulphation and therefore of activating chemicals that require sulphation for genotoxicity.

In this paper, we examine the activation of estragole and 1′-hydroxyestragole in the B-13 and B-13/H cells; examine the effect of SULT inhibitors on genotoxicity and screen for the presence of *N*^2^*-*(*trans*-isoestragol-3′-yl)-2′-deoxyguanosine (E-3′-*N*^2^-dGuo) adducts. We demonstrate for the first time that B-13/H cells express functional SULT2B1 activity and that hepatocytes and cholangiocytes express this isoform in both rat and human liver.

## 2 Materials and methods

### 2.1 Isolation of cholangiocytes

Rat liver cholangiocytes were isolated essentially as a mixed population with fibroblasts and a minor progenitor component cell population by pronase digestion of the biliary tree remaining after a two-step collagenase digestion of rat liver, see Probert et al. (2015). A relatively pure population of human liver cholangiocytes were isolated using an immune-bead approach (which is not suitable for rat cholangiocytes). In brief, liver (typically 1–2 cm^2^) was diced (1–2 mm^2^) in a sterile Petri dish using a sterile scalpel blade and then transferred to 5 ml of 10 mg/ml collagenase A and 2 ml of 2 mg/ml DNase I. The volume was made up to 50 ml with pre-warmed 1x PBS (137 mM NaCl, 2.7 mM KCl, 10 mM phosphate pH 7.4) and incubated at 37 °C for 30–45 min. The digest was then filtered through Nybolt mesh and centrifuged at 600 × *g* for 5 min. The supernatant was discarded and the cell pellets were re-suspended in 1x PBS and centrifugation repeated twice with a small amount of DNase I added at each re-suspension. Cholangiocytes were initially semi-purified using density gradient centrifugation. Percoll (GE Healthcare) was diluted 9:1 (v/v) with 10x PBS (1.37 M NaCl, 27 mM KCl, 100 mM phosphate pH 7.4). This stock was diluted 1:2 (v/v) with 1x PBS and 3 ml was layered onto 3 ml of stock diluted 1:0.3 (v/v) with 1x PBS in a 13 ml sterile falcon tube. Three ml of cells suspension was then layered onto the top of each percoll layer followed by centrifugation at 670 ×* g* for 30 min at 80% acceleration and 0% deceleration in a swing out rotor. The lower percoll/percoll interface was enriched in cholangiocytes and these were collected, added to 3 volumes of 1x PBS, pelleted by centrifugation at 600 ×* g* for 5 min and re-suspended in 9 ml of 0.1% BSA in 1x PBS. This suspension was then subjected to immuno-magnetic purification using HEA-125 Dynabeads (Thermofisher, UK) essentially according to the manufacturer’s instructions. Cells were normally used directly for analysis, however a proportion of cells were routinely cultured in 1:1 [v/v] DMEM:Hams F12 medium supplemented with 10% (v/v) FBS, 2 mM glutamine, 100 U/ml penicillin, 100 μg/ml streptomycin, 10 ng/ml epidermal growth factor (EGF), 0.248 IU/ml Insulin, 2 μg/ml hydrocortisone, 10 ng/ml cholera toxin, 2 nM tri-iodo-l-thyronine and 5 ng/ml hepatocyte growth factor (HGF) to assess purity. Human liver tissue was prepared from the margins of tissue removed from patients having a resection for both benign and malignant tumours. Tissue was obtained with patient consent and with approval of the Newcastle & North Tyneside 2 Research Ethics Committee.

### 2.2 Cell culture

B-13 cells were routinely cultured in low glucose (1 g/L) Dulbecco’s Modified Eagle’s Medium (DMEM) containing 10% (v/v) fetal calf serum (FCS), 100 units/ml penicillin, 100 μg/ml streptomycin and 0.584 g/L l-glutamine at 37 °C in an humidified incubator gassed with 5% CO_2_ in air. B-13 cells were sub-cultured every 2–3 days by trypsinization or differentiated to B-13/H cells via treatment with 10 nM dexamethasone (DEX) for 14 days, with media changes every 2–3 days.

### 2.3 Comet assay

Genotoxicity, expressed as the mean olive tail moment (OTM), was calculated from 50 individual cells using Autocomet software (Tritek Corp., Summerduck, Virginia) essentially as previously outlined (Probert et al., 2014a). Cells were pre-treated with potential inhibitors for 6 h prior to exposure to estragole or 1′-hydroxyestragole for 24 h and analysis. To control for any effects on cell viability and proliferation (which can give false positive results), compounds were separately tested for cytotoxicity or DNA synthesis as described (Mosesso et al., 2012, Probert et al., 2014a).

### 2.4 DNA adducts

Cells were pre-treated with potential inhibitors for 6 h prior to exposure to estragole or 1′-hydroxyestragole for 24 h. Cells were then scraped into ice-cooled PBS (137 mM NaCl, 2.7 mM KCl, 10 mM phosphate pH 7.4), centrifuged at 16,000 × *g* for 60 s and snap frozen in liquid nitrogen. DNA was purified from the cell pellets using a Get pure DNA Kit-Cell protocol (Dojindo Molecular Technology Inc., Kumamoto, Japan) following the manufacturer’s instructions for tissue. The DNA was re-suspended in 100 μL MilliQ water and the yield and purity determined by measuring the absorbance ratio at 260 nm and 280 nm using a molar extinction coefficient for double stranded DNA of 50 (L/mol/cm). DNA samples with an absorbance ratio of 1.8–2.0 were considered sufficiently pure. Digestion of DNA, and quantification of E-3′-N2-dGuo was performed as previously described using LC/MS-MS (Paini et al., 2010).

### 2.5 mRNA analysis

Total RNA was isolated from cultured cells or rat tissues (male adult Sprague-Dawley) using TRIzol (Invitrogen, Paisley, UK) following the manufacturer’s instructions. SYBR green was used for quantitative RT-PCR using a 7500 Fast Applied Biosciences thermocycler essentially as previously outlined (Probert et al., 2014a). The primer sequences are given in Table 1 and single amplicons determined by ethidium bromide agarose gel electrophoresis as previously outlined (Leel et al., 2004).

### 2.6 Western blotting

Western blotting was performed essentially as previously described (Marek et al., 2005). The antibodies used in these studies were rabbit anti-SULT1A1 (av49134, Sigma Chemical Co., Poole, UK), rabbit anti-SULT1B1 (ab89707, Abcam, Cambridge, UK), goat anti-SULT2B1 (sc-46542, Santa Cruz, Heidelberg, Germany), mouse anti-SULT4B1 (sc-374545, Santa-Cruz), rabbit anti-PAPSS1 (ab155600, Abcam) and rabbit anti-PAPSS2 (ab155588, Abcam). The anti-SULT2B1 antibody is reported by the manufacturer to cross-react with both rat and human SULT2A1a and SULT2A1b isoforms. Detection was carried out using the relevant anti-IgG horseradish peroxidase (HRP)—conjugated secondary antibody and ECL-based chemiluminescent detection (GE Healthcare, Amersham, UK).

### 2.7 Estragole metabolism in cultured cells

Estragole metabolism was examined by incubating cells with 1 mM estragole in HEPES/HBSS (0.14 M NaCl, 5.4 mM KCl, 0.34 mM Na_2_HPO_4_ 12H_2_O, 0.44 mM KH_2_PO_4_, 5.6 mM glucose, 6 mM HEPES, 4 mM NaHCO_3_ and 1 mM CaCl_2_) for the indicated time points. Estragole and its metabolites were then analysed by HPLC with UV detection essentially as previously described (Punt et al., 2008). In brief, separation was carried out using a Hypersil ODS C18, 5 μm (150 × 4.6 mm) column (Thermo Scientific, UK) and a gradient mobile phase (flow rate 1 ml/min) constituting H_2_O (buffer A) and acetonitrile (buffer B) with buffer B constituting 0–100% over 20 min. Eluted analytes were monitored at 225 nm between 0 and 30 min, with authentic estragole and 1′-hydroxyestragole used to confirm the identity of their peaks in chromagrams. Standard curve and sample quantification was based on peak area using LCSolution software (Shimadzu).

### 2.8 Hydroxycoumarin glucuronidation and sulphation assay

For the S9 incubations, 40 μg of human S9 extract was incubated with 25 μM 7-hydroxycoumarin and 0.1 mM PAPS in 0.1 M Tris pH 7.4 in a total reaction volume of 100 μl. After a 10 min incubation at 37 °C, reactions were terminated by addition of 25 μl of ice-cooled acetonitrile. Samples were then centrifuged at 16,000 ×* g* for 5 min and the supernatant retained for analysis. Cellular activity was determined by incubating cells in 25 μM 7-hydroxycoumarin in HEPES/HBSS for the indicated time points. Glucuronidated, sulphated and parent 7 hydroxycoumarin concentrations were determined by HPLC analysis essentially as previously described (Alhusainy et al., 2010) using a Shimadzu system HPLC machine and by comparison to authentic standards (obtained from Sigma Chem., Co., Poole, UK). Separation was carried out using a Hypersil ODS C18, 5 μm (250 × 4.6 mm) column (Thermo Scientific, UK) using a gradient created by Buffer A (0.1% (v/v) aqueous acetic acid) and Buffer B (0.1% (v/v) acetic acid in acetonitrile). Flow rate was 1 ml/min and gradient was as follows: Buffer B was increased from 0 to 20% in 2 min, then 20–40% in 8 min, during which the analytes eluted. A subsequent 40–100% increase in 2 min followed by returning to 0% buffer B in 1 min and running for 6 min allowed for re-equilibration. Standards: 7-hydroxycoumarin (7HC), 7HC-sulphate and 7HC-glucuronide (dissolved in DMSO) were diluted in PBS to create working standards at 10 μM and then subsequently doubly diluted to create a standard curve. Acetonitrile was added to all standards and samples to give a final concentration of 20% v/v (i.e. 100 μl sample + 25 μl acetonitrile), prior to centrifugation at 20,000 × *g* for 5 min and injection (20 μl). Eluted analytes were monitored at 280 nm between 2 and 10 min. Standard curve and sample quantification was based on peak area using LCSolution software (Shimadzu). Conjugation rates were normalised to protein concentation as determined by the Lowry assay.

### 2.9 SULT assay

Cell sample extracts were prepared in 5 mM phosphate buffer (pH 6.5) supplemented with 10 mM dithiothreitol. Human liver was obtained via the Newcastle Hepatopancreatobiliary Research Tissue Bank with full ethical approval and donor consent. S9 extract (from donor NHL47) was prepared by homogenising washed liver tissue in ice cold TKMS buffer (50 mM Tris; 25 mM KCl, 5 mM MgCl_2_ and 250 mM sucrose—pH 7.5.) using a dounce homogeniser, filtering through bolting cloth and centrifugation at 12,000 × *g* for 20 min at 4 °C. The supernatant (S9) was retained and used for subsequent experiments. To determine SULT activities using a variety of substrates, a reaction cocktail was prepared consisting of 50 mM phosphate buffer (pH 6.5), 24 mM dithiothreitol, 5 mM MgCl_2_ and 1.28 μM [^35^S]-PAPS (PerkinElmer, Seer Green, UK). 50ul pf the reaction cocktail was added to cell or S9 extracts in 100μl 5 mM KH_2_PO_4_ and 10 mM dithiothreitol (pH 6.5) with substrates or inhibitors as required and incubated for 30 min at 37 °C. Reactions were stopped through the addition of 100μl of a 1:1 (v:v) solution of 100 mM barium hydroxide and 100 mM barium acetate. Free PAPS was precipitated by addition of 50μl of 100 mM zinc sulphate, centrifugation at 16,000 × g for 3 min, addition of 50 μl of 100 mM barium hydroxide and 50 μl of 100 mM zinc sulphate and centrifugation at 16,000 × *g* for 3 min. 300 μl of the supernatant was transferred to 5 ml of scintillant (Optiphase HiSafe 3, PerkinElmer) and ^35^S product radioactivity determined using a Tri-Carb 2700TR scintillation counter.

### 2.10 Immunohistochemistry

Liver was formalin-fixed and processed for immunohistochemistry essentially as previously outlined (Probert et al., 2014b). The same primary antibodies used for Western blotting were used for immunohistochemistry. For SULT2B1, after antigen retrieval and before serum blocking, tissues were blocked with avidin, then biotin, for 20 min respectively (Vector Laboratories, Peterborough, UK). The secondary antibody used was biotinylated anti-goat (Sigma) and following secondary antibody incubation and washing tissues were incubated with Vectorstain ABC reagent (Vector Laboratories) for 30 min prior to detection of antibody binding.

### 2.11 Statistics

Significance was tested with by the Student’s unpaired *t*-test (for testing between 2 groups) or by ANOVA followed by the Bonferroni–Holm post hoc test (for testing multiple groups). Significance was achieved where *p* < 0.05.

## 3 Results

### 3.1 1′-hydroxyestragole genotoxicity in B-13/H cells is inhibited by general SULT inhibitors

**Fig. 2:**
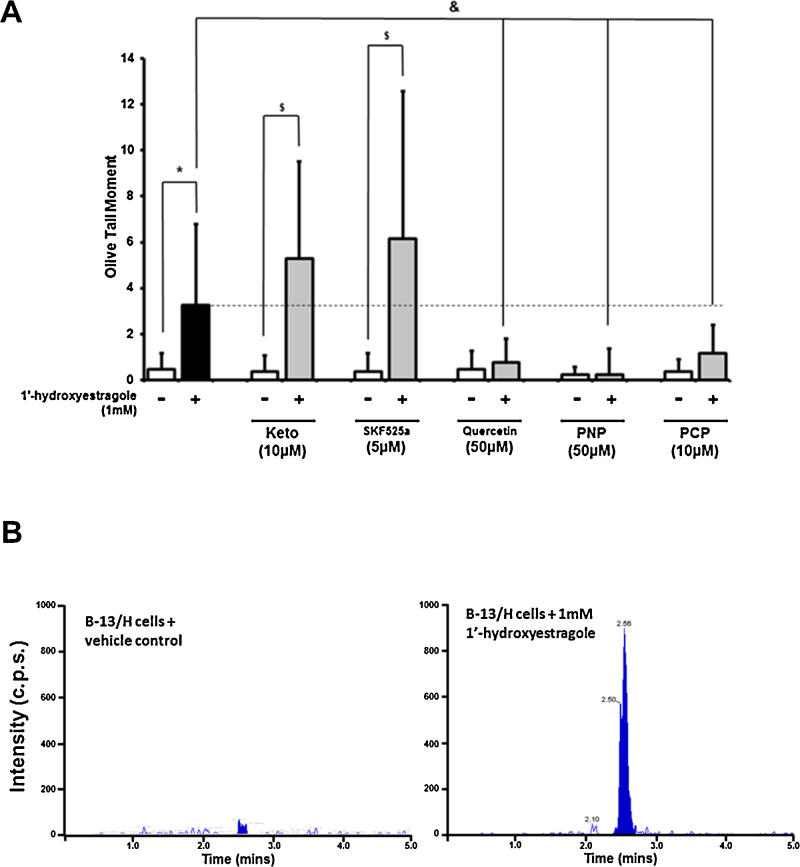
1′-hydroxyestragole but not estragole caused SULT-dependent DNA damage in B-13/H cells. A. DNA damage in B-13/H cells treated with cytochrome P450 and SULT inhibitors in combination with 1′-hydroxyestragole. Cells were pre-treated with the indicated inhibitors for 6 h prior to addition of vehicle or 1 mM 1′-hydroxyestragole. *, ^$^ and ^&^indicate significant difference. B. Chromatograms of N2-(trans-isoestragol-3′-yl)-2′-deoxyguanosine DNA-related adduct measurement from lysates of B-13/H cells treated for 24 h with vehicle or 1′-hydroxyestragole. All results are typical of at least 3 separate experiments.

**Fig. 3:**
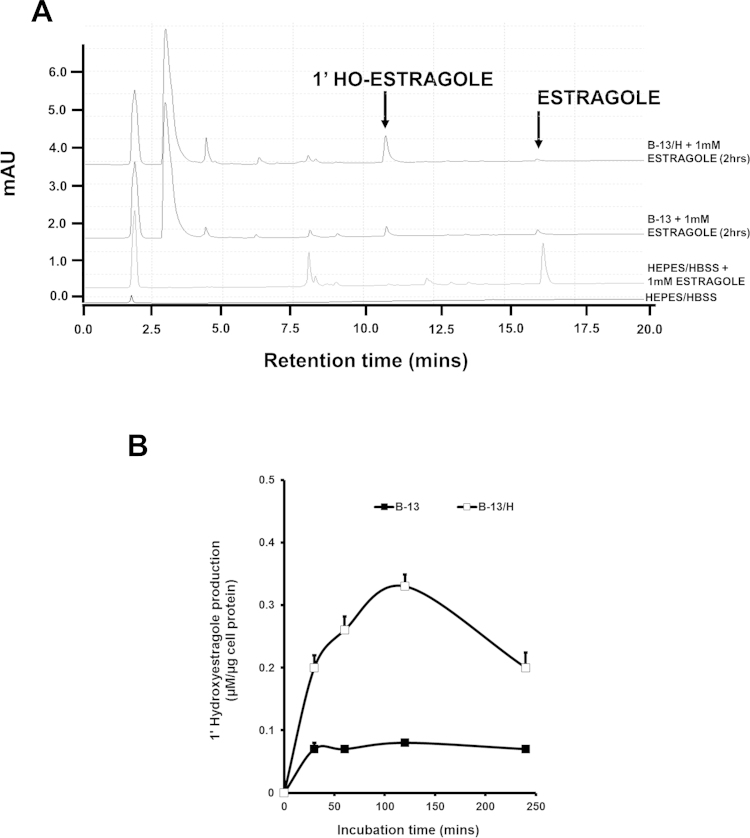
Metabolism of estragole to 1′-hydroxyestragole by B-13 and B-13/H cells. A, HPLC separation of estragole and 1′-hydroxyestragole. Authentic estragole and 1′-hydroxyestragole were subjected to HPLC to determine the retention time of their peaks, and are indicated. B. Production of 1′-hydroxyestragole by B-13 and B-13/H cells over 4 h. Media samples were taken at the indicated time points and 1′-hydroxyestragole formation determined by HPLC. Results were normalised to cell protein concentration. The average B-13/H protein content in these studies was 80 μg/well. Data are the mean and standard deviation of 3 replicates from the same experiment, typical of 3 separate experiments.

Exposing B-13 or B-13/H cells to estragole at concentrations up to 1 mM did not result in a detectable increase in DNA damage as determined by comet assays. In contrast, exposing B-13/H cells to 1′-hydroxyestragole resulted in a dose-dependent increase in DNA damage at concentrations greater than 100 μM, whereas B-13 cells where unaffected (Fig. 1B–D). Pre-treating B-13/H cells with cytochrome P450 inhibitors ketoconazole or SKF525a lead to a trend of increased DNA damage whereas pre-treatment with general SULT inhibitors quercetin (De Santi et al., 2002), ρ-nitrophenol (Eaton et al., 1996) or pentachlorophenol (Seah and Wong, 1994) significantly reduced DNA damage caused by 1′-hydroxyestragole (Fig. 2A). Fig. 2B indicates that exposing B-13/H cells to 1′-hydroxyestragole resulted in detectable levels of the predicted E-3′-*N*^2^-dGuo DNA adduct associated with sulphation activation of estragole. There was no evidence of adduct formation with high 1 mM concentrations of estragole (data not shown). Examining the metabolism of estragole demonstrated that B-13/H cells were more active than B-13 cells in generating 1′ hydroxyestragole (Fig. 3). However, the levels of 1′-hydroxyestragole did not exceed at its maximum (at 2 h) approximately 26 μM in the medium of B-13/H cultures (Fig. 3B).

These data suggest that B-13/H cells do not convert estragole to 1′-hydroxyestragole sufficiently to give rise to detectable levels of DNA damage but are capable of conjugating 1′-hydroxyestragole with sulphate catalysed by a SULT enzyme(s) leading to the production of 1′-sulphoxyestragole, its reactive carbocation and genotoxicity.

### 3.2 B-13/H cells do not express functionally significant levels of SULT1A1

A previous RT-PCR study indicated that a variety of genes defined as encoding phase II enzymes (SULTs UDPGTs and GSTs) are expressed in B-13/H cells at the level of mRNA (Probert et al., 2014a). To determine the levels of expression of SULTs relative to normal rat liver, quantitative expression levels of SULTs and PAPSSs identified as being expressed in B-13/H cells were examined quantitatively at the level of mRNA and protein in B-13 cells, B-13/H cells, male rat liver and male rat pancreas [note, that B-13 cells have been shown to be derived from male rats (Fairhall et al., 2013)].

**Fig. 4:**
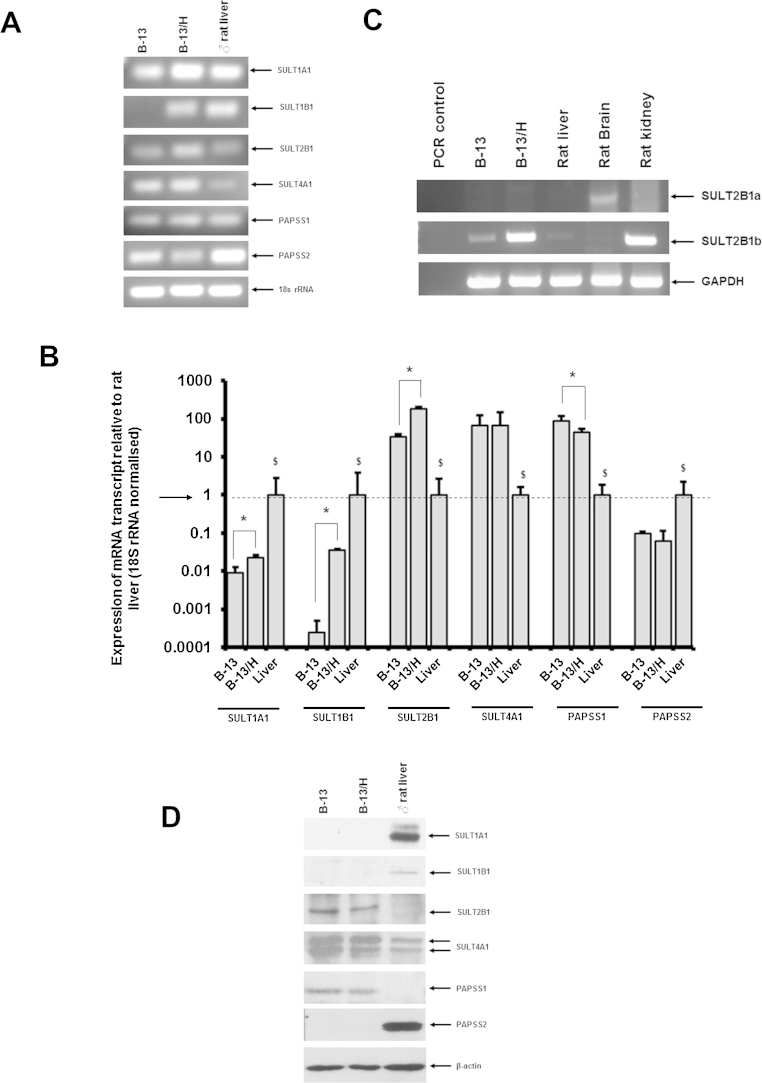
B-13 and B-13/H cells express SULT transcripts and SULT2B protein. A. RT-PCR of indicated transcripts in B-13 and B-13/H cells and rat liver. DNA samples from the qRT-PCR runs of B were separated and visualised by agarose gel electrophoresis. B. qRT-PCR of indicated transcripts in B-13 and B-13/H cells and rat liver. Expression of transcripts was normalised to 18S rRNA expression and expressed relative to rat liver. ^*^Significantly different level of expression between B-13 and B-13/H cells; ^$^ Significantly different level of expression between rat hepatocytes and both B-13 and B-13/H cells C. RT-PCR for SULT2B1a and SULT2B1b mRNA transcripts in indicated samples. D. Western blot for indicated proteins in B-13 and B-13/H cells and rat liver. Results are typical of at least 3 separate determinations.

Fig. 4A demonstrates that the quantitative RT-PCR conditions amplified a single amplicon of the correct size for each transcript. For all SULT transcripts, there was an induction in the expression of mRNAs when B-13 cells were differentiated into B-13/H cells, except for SULT4A1 which remained unaffected (Fig. 4B). In comparison to rat liver however, expression of SULT1A1 and SULT1B1 mRNAs were between 10 and 100 fold lower than the levels expressed in rat liver (Fig. 4B). In contrast, both B-13 and B-13/H cells expressed SULT2B1 and SULT4A1 mRNAs between 10 and 100 fold higher than the levels expressed in rat liver (Fig. 4B).

The SULT2B1 gene produces two transcripts, SULT2B1a and SULT2B1b (and both transcripts are predicted to be amplified by the primer pair used in qRT-PCR). Fig. 4C indicates the SULT2B1 mRNA expression is likely composed entirely of SULT2B1b transcripts, since only the SULT2B1b transcript was detectable by RT-PCR using variant-specific primers (see Supplementary Fig. 1). Fig. 4B indicates that PAPSS1 mRNA transcripts where expressed similarly in both B-13 and B-13/H cells at greater than 10 fold the levels detected in rat liver whereas PAPSS2 mRNA levels were greater than 10 fold lower. Western blotting confirmed that both SULT2B1 and SULT4A1 proteins were readily detectable in B-13 and B-13/H cells (Fig. 4D).

These data indicate that both B-13 and B-13/H cells express a restricted set of SULT and PAPSS proteins and therefore suggest a potential capacity for sulphation of a limited set of substrates. However, the expression pattern of these genes in B-13/H cells is not reflective of the pattern observed in male rat hepatocytes and notably, SULT1A1–determined to be the major isoform mediating the conjugation of 1′-hydroxyestragole that leads to the generation of a genotoxic carbocation (Herrmann et al., 2012) – was expressed at very low to undetectable levels in B-13 and B-13/H cells.

### 3.3 The SULT1A1 substrate 7 hydroxycoumarin is not sulphated by B-13 or B-13/H cells

**Fig. 5:**
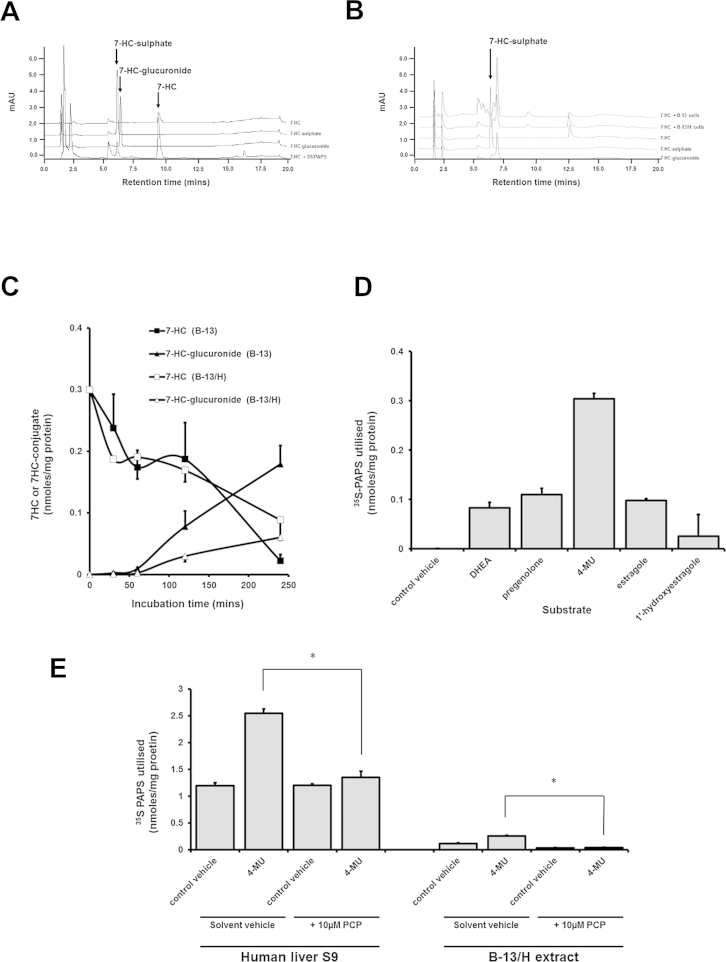
B-13/H but not B-13 cells show SULT2B enzyme activity. A. HPLC separation of 7-HC, 7-HC- glucuronide and 7-HC- sulphate. B. HPLC separation of S9 7-HC incubation and 7-HC standards. C. Metabolism of 7-HC by B-13 and B-13/H cells over 4 h. Media samples were taken at the indicated time points and 7-HC-sulphate and 7-HC-glucuronide formation determined by HPLC. Results were normalised to cell protein concentration. D. ^35^S PAPS utilisation in B-13/H cell lysates following addition of the indicated substrates. Results were normalised to protein concentration E. ^35^S PAPS utilisation in human liver S9 and B-13/H cell lysate incubated with 4-MU alone or in combination with PCP. *indicates significant difference. All results are typical of at least 3 separate experiments.

7 Hydroxycoumarin is primarily sulphated by SULT1A1 as well as being a substrate for UDP-glucuronyl transferases (UDP-GTs). To confirm that B-13 and B-13/H cells express low levels of SULT1A1, cells were incubated with 7 hydroxycoumarin and medium analysed for the presence of glucuronidated and sulphated conjugates. Fig. 5A–C show that 7 hydroxycoumarin glucuronide was readily generated in both B-13 and B-13/H cells whereas the sulphated conjugate was not detected despite greater than 60% disappearance of 7 hydroxycoumarin during the time course of incubations. This disappearance and appearance of glucuronide conjugate demonstrates that the absence of a sulphated conjugate was not associated with a lack of cellular uptake.

In contrast, sulphation of SULT2B1 substrates-DHEA, pregnenolone and 4MU was detected in B-13/H extracts, in addition to estragole (which was hydroxylated in the extract, data not shown) and 1′-hydroxyestragole (Fig. 5D). Despite similar expression of SULT mRNA transcripts and proteins in B-13 and B-13/H cells, functional activity towards all these substrates in B-13 cells was not observed (data not shown).

Fig. 5E demonstrates that in comparison to human liver S9 extract, total sulphation of 4MU in B-13/H cell extracts remains around 10% of the levels observed in liver extracts. However, sulphation is inhibited by the broad spectrum SULT inhibitor pentachlorophenol (PCP) in both S9 and B-13/H extracts, confirming that B-13/H cells express functional SULT2B1 activity.

### 3.4 Cholangiocytes express SULT2B1, suggesting cholangiocytes are a potential target for estragole genotoxicity

Since the most common tumour observed in male rats exposed to estragole was multiple cholangiocarcinomas (Miller et al., 1983, Bristol, 2011), we hypothesised that SULT expression in cholangiocytes may play a role in this process. Immunohistochemical staining for SULTs indicated that SULT1A1 expression was restricted to hepatocytes, with the highest levels present in hepatocytes in the centrilobular region of the lobule in rat liver. SULT1A1 expression was also high in human hepatocytes although expression was patchy throughout the lobule, expression appeared distinct from rat in that levels were high in hepatocytes in the centrilobular and periportal regions of the lobule (Supplementary Fig. 2). SULT1B1 expression in rat liver was highest in cholangiocytes, with some expression in the hepatocytes in the centrilobular region around the central vein. In contrast, SULT1B1 expression in human liver was not apparent in cholangiocytes and expression in hepatocytes was highest in the periportal region (Supplementary Fig. 3).

**Fig. 6:**
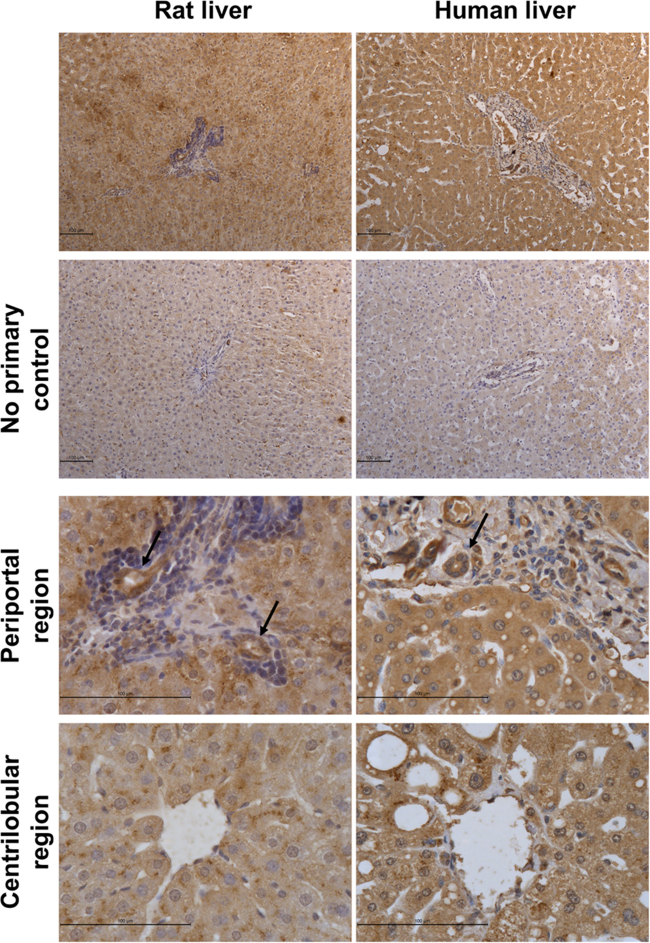
Rat and human cholangiocytes express SULT2B1. Photomicrographs of rat and human liver immunostained for SULT2B1. Results are representative of at least 3 separate rat and human liver samples. Human liver sample shown is from donor NHL17. Arrows indicate bile ducts lined with cholangiocytes. Immunoreactivity was inhibited by co-incubation with antigen blocking peptide sequence (see Supplementary Fig. 4).

**Fig. 7:**
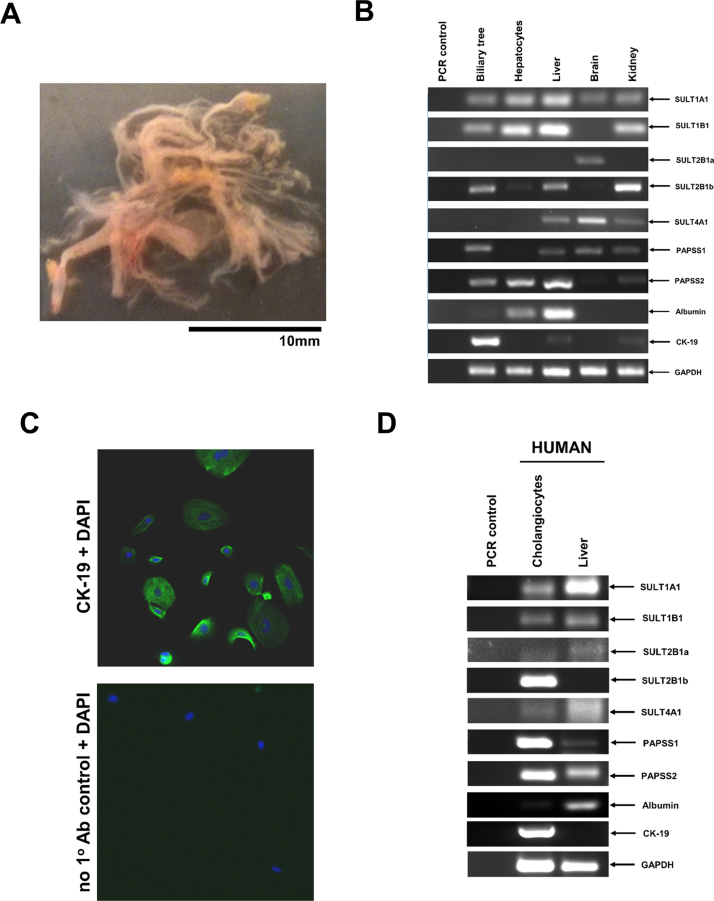
Rat and human cholangiocytes express SULT2B1b mRNA transcripts. A. Photomicrograph of remaining biliary tree after extensive digestion of liver tissue. B. RT-PCR analysis for the indicated transcript in rat tissues. Data are typical of determinations from at least 3 separate tissue donors. C. Immunocytochemical analysis for the ductal marker cytokeratin 19 (CK-19, green) in human cholangiocyte preparations after culture of cells for 24 h. Nuclei are stained with DAPI (blue). Data typical of 6 separate isolations. D. RT-PCR analysis for the indicated transcript in human cells. Data are typical of determinations from at least 3 separate tissue donors. (For interpretation of the references to colur in this figure legend, the reader is referred to the web version of this article.)

Expression of SULT2A1 in both rat and human showed a similar panlobular expression pattern in hepatocytes. In addition, expression of SULT2B1 was high in cholangiocytes in both rat and human liver sections (Fig. 6). These data were underpinned by the observation that addition of peptide to which the anti-SULT2B1 antibody binds, blocked antibody binding to liver sections indicating that stain intensity is associated with specific binding of anti-SULT2B1 to SULT2B1 alone (Supplementary Fig. 4). Furthermore, RT-PCR analysis for transcripts from isolated rat liver biliary tree confirmed enriched expression of the SULT2B1b transcript (Fig. 7A–B). Isolation of a pure population of human cholangiocytes using an antibody-bead based approach (Fig. 7C, note this procedure is not successful when applied to rat cells) confirmed enriched expression of the SULT2B1b transcript in human cholangiocytes (Fig. 7D).

Western blotting (Fig. 4D) suggested that rat liver expressed SULT4A1 proteins. However, immunohistochemical staining for SULT4A1 expression was unsuccessful on both rat and human liver sections (data not shown), suggesting the antigen(s) recognised by the anti-SULT4A1 antibody was destroyed by tissue fixation and/or processing.

## 4 Discussion

The initial aim of this work was to determine whether the B-13/H cell platform was able to metabolise a phase II enzyme-activated genotoxin (estragole) and therefore provide a simple in vitro genotoxicity screening system. The data demonstrate that there was little evidence for genotoxicity when cells were exposed to estragole, likely because estragole is extensively metabolised to non-genotoxic metabolites as previously reported (Anthony et al., 1987, Luo and Guenthner, 1994, Luo and Guenthner, 1995, Punt et al., 2008); B-13/H cells express high levels of a number of cytochrome P450s (Marek et al., 2003, Probert et al., 2014a) and as reported herein, the concentrations of 1′-hydroxyestragole generated from estragole were low. However, addition of the proximate SULT1A1-activated genotoxic metabolite 1′-hydroxyestragole to B-13/H cells resulted in a clear dose-dependent increase in DNA damage in comet assays, confirmed by detection of E-3′-*N*^2^-dGuo adducts. DNA damage was inhibited by general SULT inhibitors, supporting a role for SULTS in the activation of 1′-hydroxyestragole in B-13/H cells. Addition of general cytochrome P450 inhibitors had a tendency to increase the genotoxicity of 1′-hydroxyestragole suggesting that cytochrome P450s also metabolise 1′-hydroxyestragole to non-genotoxic metabolites in B-13/H cells.

Although B-13/H cells are a good model for rat hepatocytes in terms of cytochrome P450 expression, B-13/H cells did not express significant levels of the SULT1A1 as determined by qRT-PCR, Western blotting and 7 hydroxycoumarin conjugation with sulphate. In this respect therefore, B-13/H cells appear functionally null for SULT1A1. Since B-13/H cells were able to sulphate 1′-hydroxyestragole in cell extracts, these observations suggest that 1′-hydroxyestragole was conjugated by other SULTs.

Progenitor cells in the liver are bi-potential, forming both hepatocytes and cholangiocytes. Although the B-13 progenitor cell is thought to derive from the embryologically closely-related pancreas (Probert et al., 2015), recent work has identified that the B-13 cell can express some cholangiocyte markers (Al-Adsani et al., 2010). The B-13/H cells may therefore be more reflective of rat cholangiocytes – rather than hepatocyte – in terms of SULTs expression.

Physiologically-based biokinetic modeling of estragole metabolism in the rat suggests that at low doses of estragole, there is primarily detoxication through formation of 4-allylphenol, occurring mainly in the lung and kidney. Saturation of this metabolic pathway leads to increased formation of the proximate carcinogenic metabolite 1′-hydroxyestragole in the liver and to a relative increase in formation of 1′-hydroxyestragole glucuronide and 1′-sulphooxyestragole, the latter being the ultimate carcinogenic metabolite of estragole (Punt et al., 2008). 1′-hydroxyestragole has been shown to be metabolised almost exclusively to 1′-sulphooxyestragole by SULT1A1 in man (Herrmann et al., 2012). The data in this paper suggests that in rat, other SULTs such as SULT2B1b, may be capable of activating 1′-hydroxyestragole. This paper also demonstrates that rat and human cholangiocytes express SULT2B1, but not SULT1A1. Since the most common tumour observed in male rats exposed to estragole was multiple cholangiocarcinomas (Miller et al., 1983, Bristol, 2011), the genotoxic metabolic pathway in rats may be significantly dependent on activation by SULT2B1. It is of interest to note that for the related alkenylbenzene methyleugenol, human SULT2B1b did not seem to be involved in its bioactivation (Herrmann et al., 2012). This suggests that depending on the alkenylbenzene, species or tissue under study, the SULT enzymes involved in bioactivation of the alkenylbenzenes may vary. This could imply that the relevance of the cholangiocarcinomas observed in estragole-exposed rats may need to be reconsidered for human risk assessment.

## Conflict of interest

The authors declare that they have no conflict of interest.

## Figures and Tables

**Table 1:** DNA oligonucleotide sequences employed in qRT-PCR and RT-PCR.

Oligo ID	5'-3' sequence	Comments
qRT-PCR		

rSULT1A1US	ACACATCTGCCCCTGTCCT	Will amplify 77 bp cDNA fragment of NM_031834.1 (Probert et al., 2014a).
rSULT1A1DS	GCATTTCGGGCAATGTAGA
rSULT1B1US	CGAGATGTTATTACCTCTAAAGTTCCA	Will amplify 88 bp cDNA fragment of NM_022513.2 (Probert et al., 2014a).
rSULT1B1DS	GAGTTTTCTTCAAGAGTTCAACACC
rSULT2B1US	CCCACCTCCCTATTGAACTG	Will amplify 70 bp cDNA fragment of NM_001039665.1.
rSULT2B1DS	CGGCCCAAGTAAATCACCT
rSULT4A1US	AAGATATGCACCGGGACCT	Will amplify 65 bp cDNA fragment of NM_031641.1.
rSULT4A1DS	ACAGGACACACCCAGGAATC
rPAPSS1US	TTGCAGTGCCTTCATTTTGA	Will amplify 60 bp cDNA fragment of NM_001106471.1.
rPAPSS1DS	AGGCACGGATAAGTTGATGAC
rPAPSS2US	ACCCTACTGGACGATGGAGT	Will amplify 119 bp cDNA fragment of NM_001106375.2.
rPAPSS2DS	CTCCGGCCTTCGTACATCAA
r18SrRNAUS	CCCGAAGCGTTTACTTTGAA	Will amplify 136 bp cDNA fragment of NR_046237.1 (Probert et al., 2014a)
r18SrRNADS	CCCTCTTAATCATGGCCTCA
**–**		
RT-PCR		
rSULT2B1aUS	TCACTGGAGAAACTGAGGCAGG	Will amplify 319 bp cDNA fragment of NM_001039665.1 (Kohjitani et al., 2006).
rSULT2B1aDS	GGAAGGCTGAAGGCACTTATGG
rSULT2B1bUS	ATGGGGCTCATTGGAGAACAG	Will amplify 307 bp cDNA fragment of XM_006229031.2 (Kohjitani et al., 2006).
rSULT2B1bDS	TGGAAGGCTGAAGGCACTTATG
rALBUMINUS	TGGTCGCAGCTGTCCGTCAGA	Will amplify 192 bp cDNA fragment of NM_134326.2
rALBUMINDS	CAGGTCGCCGTGACAGCACTC
rCK-19US	TTGGGTCAGGGGGTGTTTTC	Will amplify 446 bp cDNA fragment of NM_199498.2
rCK-19DS	CTCAAACTTGGTCCGGAAGTC
hSULT1A1US	AGCTCAGAGAACAACCCTGC	Will amplify 92 bp cDNA fragment of NM_001055.3
hSULT1A1DS	CTGAGCTCTTGGGAACCTGG
hSULT1B1US	TATGCGTAAAGGGACGGCTG	Will amplify 115 bp cDNA fragment of NM_014465.3
hSULT1B1DS	TGTGCGGAATTGAAGTGCAG
hSULT2B1aUS	TCCCTACTCTCCCTCATGGC	Will amplify 197 bp cDNA fragment of NM_004605.2 also refered to as transcript variant 1
hSULT2B1aDS	ATCCAGGTCGTGCCTGACT
hSULT2B1bUS	GGGCTTGTGGGACACCTATG	Will amplify 198 bp cDNA fragment of NM_177973.1 also referred to as transcript variant 2
hSULT2B1bDS	ATCCAGGTCGTGCCTGACT
hSULT4A1US	GGTGGTCTACTTGGTGAGCC	Will amplify 128 bp cDNA fragment of NM_014351.3
hSULT4A1DS	GGGGAGAGGTCAGTTCCTTG
hPAPSS1US	GCAGAACTGGGGAATGCAGAG	Will amplify 164 bp cDNA fragment of NM_005443.4
hPAPSS1DS	AGGCCATGCTCACAGTAGTC
hPAPSS2US	GACACCCTGCTAGATGATGG	Will amplify 115 bp cDNA fragment of NM_004670.3
hPAPSS2DS	CACCATGTGCCAGGACAAAC
hALBUMINUS	AGCTGCCTGTCTGTTGCCAAA	Will amplify 134 bp cDNA fragment of NM_000477.5
hALBUMINDS	AGGCGAGCTACTGCCCATGC
hCK-19US	CAGCTTCTGAGACCAGGGTT	Will amplify 725 bp cDNA fragment of NM_002276.4
hCK-19DS	GCCCCTCAGCGTACTGATTT
rmhGAPDHUS	TGACATCAAGAAGGTGGTGAAG	Will amplify 243 bp rat (NM_017008), human (NM_002046) or mouse (NM_008084) glyceraldehyde 3 phosphate dehydrogenase (Wallace et al., 2010b).
rmhGAPDHDS2	TCTTACTCCTTGGAGGCCATGT

## Appendix A Supplementary data

Supplementary Fig. S1 Alignment of rat SULT2B1a and SULT2B1b cDNA sequences using CLUSTAL alignment software (http://www.ebi.ac.uk/Tools/msa/clustalo/). Indication of primer hybridisation sites (arrows, with primer 5’-3’ direction indicated by arrow direction). Note that on the NCBI database, rat SULT2B1b is identical to transcript variant X1 (XM_006229031.2).

Supplementary Fig. S2 SULT1A1 is expressed in centrilobular but not periportal regions of the liver. Photomicrographs of rat (A) and human (B) liver immunostained for SULT1A1. Results are representative of at least 3 separate rat and human liver samples. Human liver sample shown is from donor NHL21.

Supplementary Fig. S3 SULT1B1 is expressed by rat cholangiocytes. Photomicrographs of rat (A) and human (B) liver immunostained for SULT1B1. Results are representative of at least 3 separate rat and human liver samples. Human liver sample shown is from donor NHL21.

Supplementary Fig. S4 Rat and human SULT2B1 immunoreactivity is blocked by co-incubation with a blocking peptide containing the anti-SULT2B1 antigenic site. Photomicrographs of rat liver (A) and human liver (B) immunostained for SULT2B1 with comparison to serial sections stained identically except for the addition of blocking peptide. Results are representative of at least 3 separate rat and human liver samples. Human liver sample shown is from donor NHL17.
