# Supplementary figures and images for: Progenitor-derived hepatocyte-like (B-13/H) cells metabolise 1′-hydroxyestragole to a genotoxic species via a SULT2B1-dependent mechanism
Source: Toxicol Lett. 2016 Jan 22;243:98–110. doi: 10.1016/j.toxlet.2015.12.010 (PMC4729325; doi:10.1016/j.toxlet.2015.12.010)

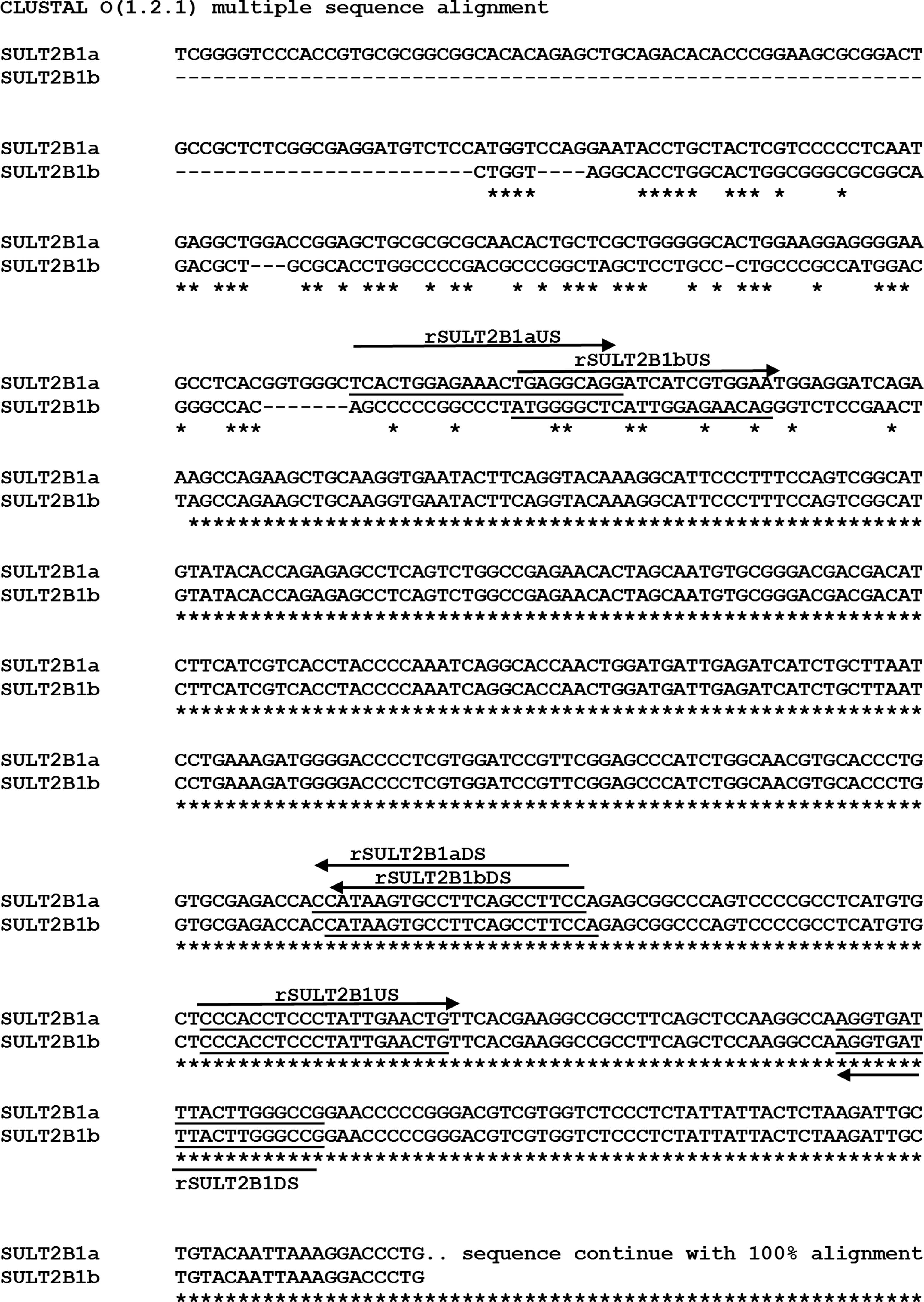

Supplement: Supplementary Fig. S1 — Alignment of rat SULT2B1a and SULT2B1b cDNA sequences using CLUSTAL alignment software (http://www.ebi.ac.uk/Tools/msa/clustalo/). Indication of primer hybridisation sites (arrows, with primer 5’-3’ direction indicated by arrow direction). Note that on the NCBI database, rat SULT2B1b is identical to transcript variant X1 (XM_006229031.2). [file mmc1.jpg]

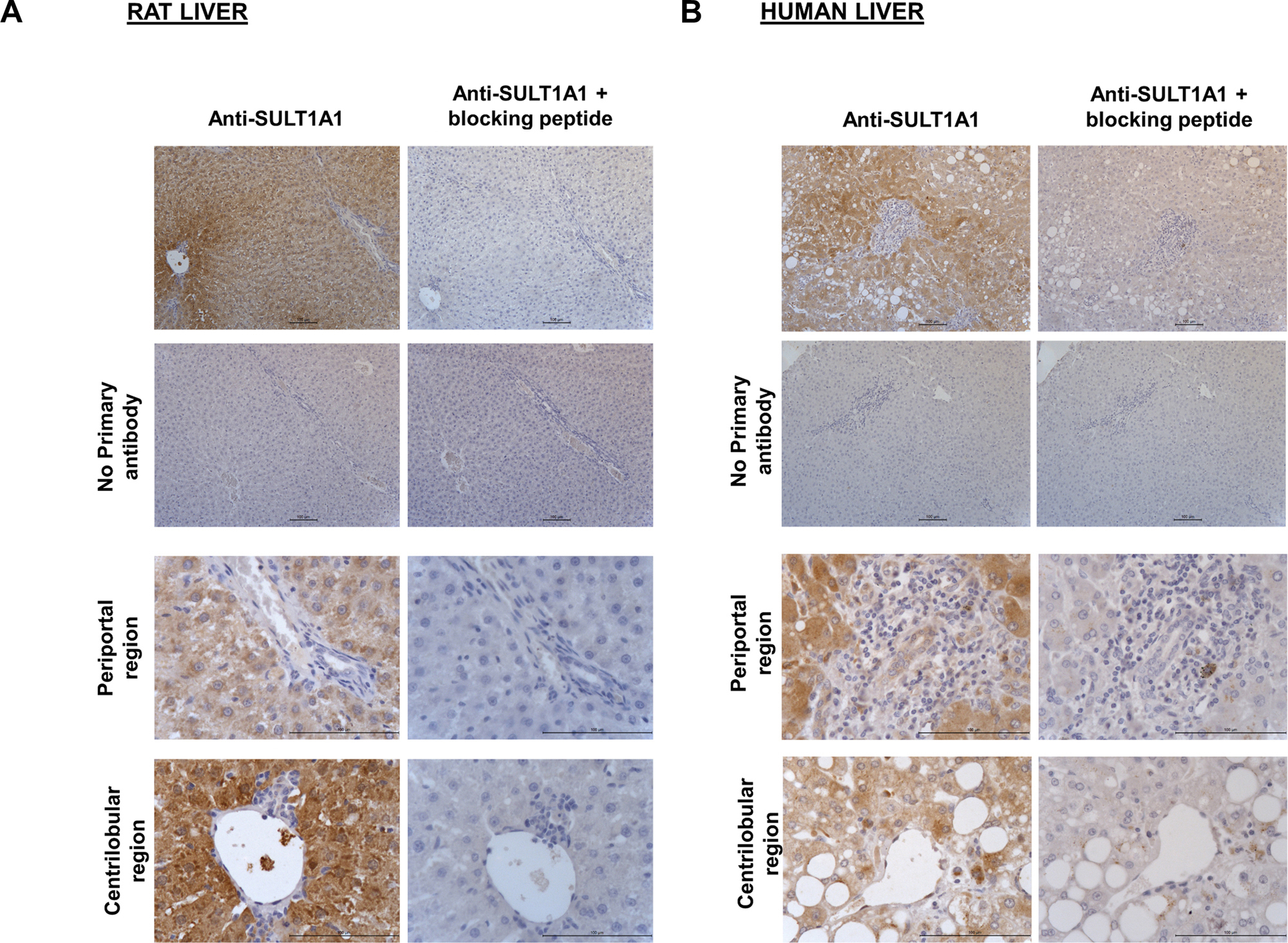

Supplement: Supplementary Fig. S2 — SULT1A1 is expressed in centrilobular but not periportal regions of the liver. Photomicrographs of rat (A) and human (B) liver immunostained for SULT1A1. Results are representative of at least 3 separate rat and human liver samples. Human liver sample shown is from donor NHL21. [file mmc2.jpg]

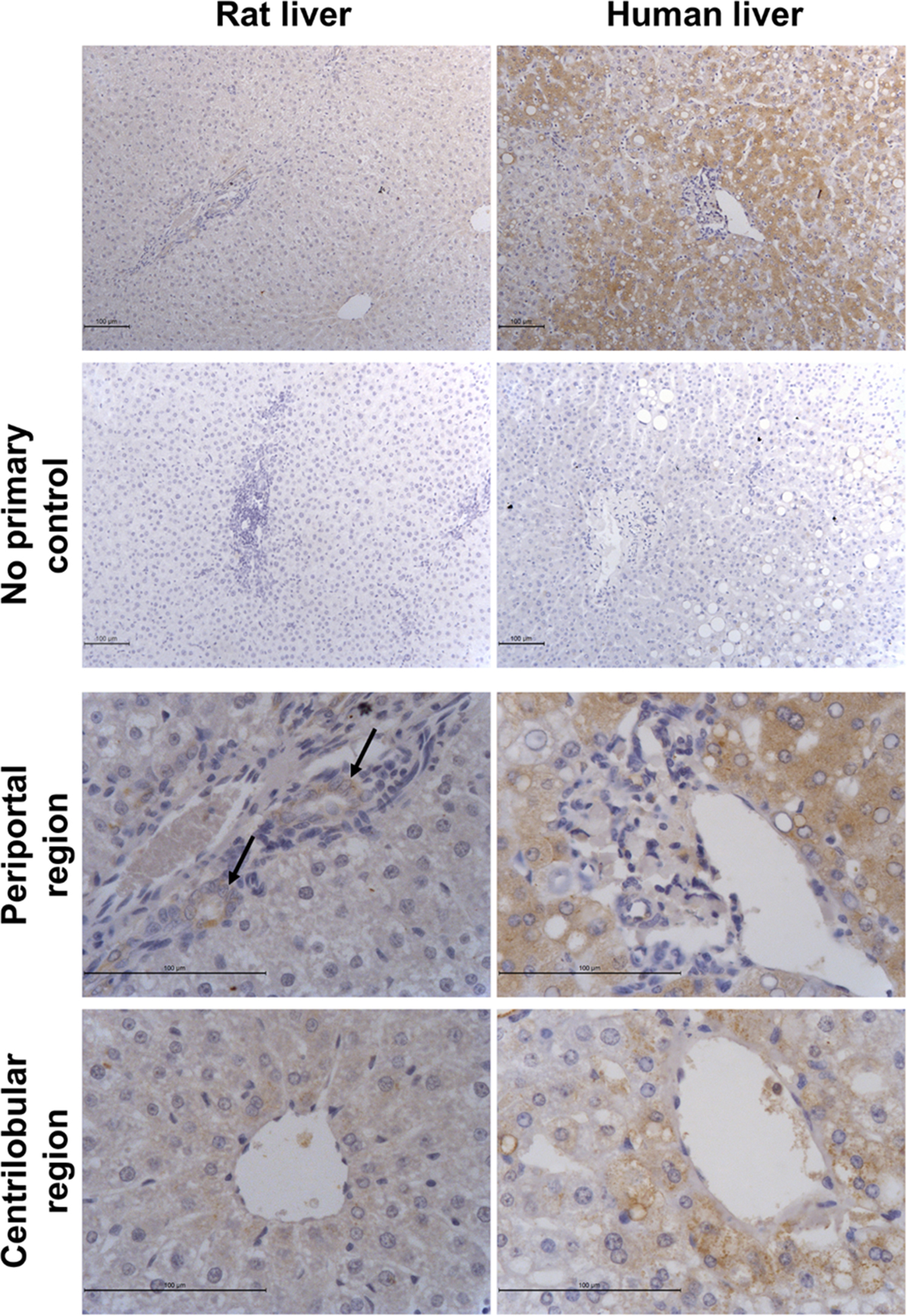

Supplement: Supplementary Fig. S3 — SULT1B1 is expressed by rat cholangiocytes. Photomicrographs of rat (A) and human (B) liver immunostained for SULT1B1. Results are representative of at least 3 separate rat and human liver samples. Human liver sample shown is from donor NHL21. [file mmc3.jpg]

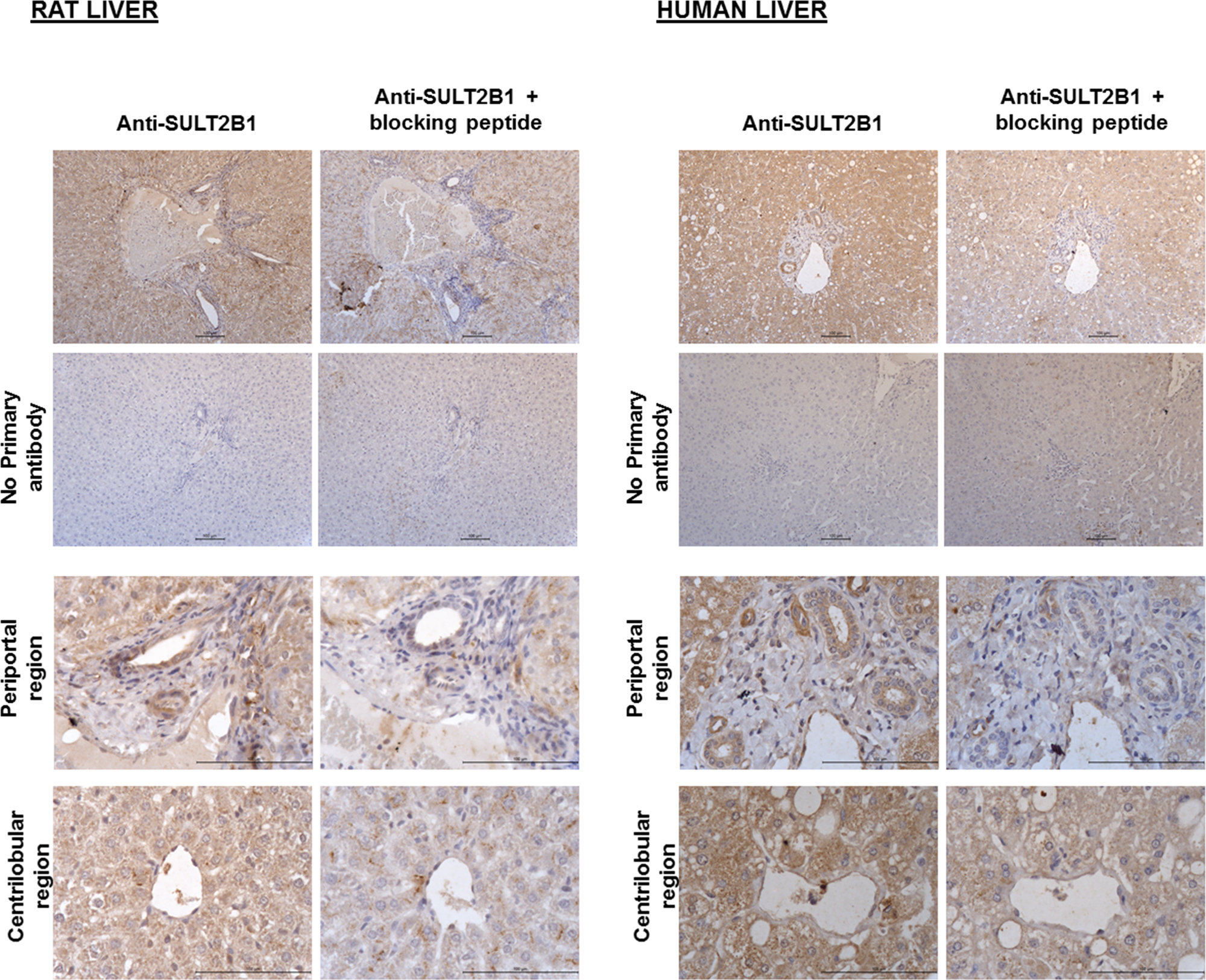

Supplement: Supplementary Fig. S4 — Rat and human SULT2B1 immunoreactivity is blocked by co-incubation with a blocking peptide containing the anti-SULT2B1 antigenic site. Photomicrographs of rat liver (A) and human liver (B) immunostained for SULT2B1 with comparison to serial sections stained identically except for the addition of blocking peptide. Results are representative of at least 3 separate rat and human liver samples. Human liver sample shown is from donor NHL17. [file mmc4.jpg]
